# The *ex vivo* effects of ethanolic extractions of black cumin seed, turmeric root, and Ceylon cinnamon bark on the human gut microbiota

**DOI:** 10.1371/journal.pone.0334824

**Published:** 2025-12-04

**Authors:** Karley K. Mahalak, Adrienne B. Narrowe, LinShu Liu, Jenni Firrman, Johanna M. S. Lemons, Pieter Van den Abbeele, Aurélien Baudot, Yuanhang Yao, Yanfang Li, Liangli Yu

**Affiliations:** 1 Dairy and Functional Foods Research Unit, Eastern Regional Research Center, Agricultural Research Service, United States of America Department of Agriculture, Wyndmoor, Pennsylvania, United States of America; 2 Cryptobiotix SA, Ghent, Belgium; 3 Department of Nutrition and Food Science, University of Maryland, College Park, Maryland, United States of America; Institute for Biological Research, University of Belgrade, SERBIA

## Abstract

Black cumin, turmeric root, and Ceylon cinnamon bark are spices that have been used for both culinary purposes and in traditional medicine practices. These spices are frequently connected with providing antidiabetic, antimicrobial, anti-inflammatory, and gastroprotective properties. However, most studies on potential health effects have not been performed in humans. Since many of the health effect claims relate to gastrointestinal health, we explored the impact of black cumin extract (BCE), turmeric root extract (TRE), and Ceylon cinnamon extract (CCE) on the human gut microbiota *ex vivo* using the SIFR^®^ technology. The impact on the gut microbiota were determined using shotgun sequencing and flow cytometry, while the health-related short-chain fatty acids (SCFA) were analyzed to assess the metabolic output. While TRE and CCE had very little effect on the gut microbiota, BCE significantly increased acetate (+ 8.7mM), butyrate (+1.3mM), and propionate (+3mM) production. This related to specific increases of *Alistipes onderdonkii*, *Alistipes shahii* and particularly *Candidatus Cibiobacter qucibialis*, CCE and TRE increased the health related *Faecalibacterium prausnitzii* and *Dysosmobacter welbionis*, respectively, with CCE also increasing *Enterococcus* and *Veillonella* species. Overall, these findings indicate these spices may have an impact on the human gut microbiome that could explain their purported health effects.

## Introduction

Black cumin seed, turmeric root, and Ceylon cinnamon sticks are common spices that are frequently used for culinary purposes but have also been used in traditional medicine for hundreds of years. The World Health Organization describes traditional medicine as the practices and knowledge based on the theories, and experiences that are indigenous to different cultures used in health maintenance and the prevention and treatment of illness [1,2]. Traditional medicine still plays a significant role in health care in many countries and communities, including China, India, and Africa [2,3]. Since the 1800s, there have been attempts to validate claims of traditional medicine and incorporate it within modern medicine via the development of scientific studies to address claims about herbal remedies [3–6].

In traditional medicine practices, black cumin seed is used to treat eczema, diabetes, inflammation, and gastrointestinal distress [7]. Research suggests that the active compounds in black cumin seed include thymoquinone, linoleic acid, oleic acid, and others. [7,8]. Constituents of black cumin seeds have been shown to have anti-inflammatory effects in rats [9] and human blood cells [10]. Essential oil from black cumin seed has an antibacterial effect on a variety of bacteria known to be resistant to antibiotics [11,12]. In two different mouse models, extracts from black cumin seeds increased specific bacterial taxa within the gut microbial community and increased the production of certain short-chain fatty acids (SCFAs), most notably butyrate and propionate [13,14].

Uses of turmeric root in traditional medicine practices include protection against asthma, as well as inflammation and gastrointestinal distress [15]. The main active ingredient of turmeric is curcumin, which has been shown to have anti-inflammatory properties and is specifically reported to decrease inflammation in the gut [16]. Turmeric root has been shown previously to modulate the gut microbiota in both humans and piglets and to increase the production of SCFAs in piglets [17,18].

Ceylon cinnamon is one of the two main types of cinnamon, both of which have been used in traditional medicine. Of the two, Ceylon cinnamon has been implicated in treating respiratory and digestive distress, via the activity of its component cinnamaldehyde [19,20]. Ceylon cinnamon has exhibited antimicrobial effects both *in vitro* on human infant gut microbiota studies and *in vivo* in mouse models [21,22].

All three spices have reported beneficial effects on gastrointestinal health, and a major mediator of gastrointestinal health is the gut microbiota [23–25]. The structure and function of the gut microbiota are shaped by diet, which includes spices and herbal remedies like those studied hereabove [26]. Some changes to the gut microbiota by diet can help protect against disease and may explain the beneficial effects of each of these three spices. However, there are very few studies on the effect of these spices on the human gut microbiota. In this study, an *ex vivo* model was employed to study the effects of ethanol extracts from each of these spices, black cumin seed, turmeric root, and Ceylon cinnamon bark, on the adult gut microbiota over a 48-hour incubation period. Following the incubation period, we used shotgun sequencing and targeted metabolomics to understand how each extract changed the structure and function of the gut microbiota.

## Materials and methods

### Preparation of spice extracts

Organic black cumin was obtained from Botanic Innovation, LLC (Wisconsin, USA), ground organic turmeric root and Ceylon cinnamon sticks were obtained from Frontier Co-Op (Iowa, USA). Each spice was ground to a particle size of < 40 mesh using a micromill (Bel-Art Products, Pequannock, NJ, USA). The materials were treated with hexane for defatting and were then dried in a fume hood overnight to remove residual hexane. After drying, each sample was extracted using 95% ethanol using the Soxhlet extraction method described previously [27]. After removal of the solvent via rotary evaporation, the dried extracts were re-ground to ensure they were a fine powder and stored at −20°C for future use.

### Untargeted metabolomics

Untargeted metabolomics were performed by Creative Proteomics on the spice extracts. For sample preparation, each extract was thawed on ice, 80% methanol was added at 8 µL/mg the raw material and two 5-mm metal balls were added to the tube. All samples were ground for 180 s at 65 Hz, twice, followed by sonication for 30 min, at 4°C. Each extract was then kept at −20°C for 1 hr, vortexed for 30 s, and centrifuged for 10 min at 12,000 rpm at 4°C. Lastly, 200 µL of supernatant was combined with 5 µL of 0.14 mg/mL DL-O-chlorophenylalanine as an internal standard, then filtered through a 0.22 µm filter.

Separation was performed using a Vanquish Flex UPLC combined with Q Exactive plus MS (Thermo Fisher Scientific, Waltham, MA, USA) which is equipped with a heated ESI source. A Hypersil gold C_18_ column (100 x 2.0 mm x 1.9 µm) was used for LC separation. The mobile phase was composed of solvent A (0.05% formic acid water) and solvent B (acetonitrile) using a gradient elution (0–1 min, 5% B; 1–12.5 min, 5%−95% B; 12.5–13.5 min, 95% B; 13.5–13.6 min, 95%−5% B; 13.6–16 min, 5% B). The flow rate of the mobile phase was 0.3 mL/min. The column temperature was maintained at 40C, and the sample manager temperature was set at 4°C.

Mass spectrometry parameters for ESI+ and ESI- modes were as follows: ESI + : heater temp 300°C; sheath gas flow rate, 45 arb; aux gas flow rate, 15 arb; sweep gas flow rate, 1 arb; spray voltage, 3.0 KV; capillary temperature, 350°C; S-Lens RF Level, 30%; ESI-: heater temp 300°C, sheath gas flow rate, 45 arb; aux gas flow rate, 15 arb; sweep gas flow rate, 1 arb; spray voltage, 3.2 KV; capillary temperature, 350°C; S-Lens RF Level, 60%. The data acquisition mode used was data-dependent acquisition (DDA).

Data preprocessing and compound identification was performed using Compound Discoverer v. 3.1(Thermo Fisher Scientific, Waltham, MA, USA). For metabolite identification, the ion peak and ion fragment information was assisted by mzVault local database (Thermo fisher Scientific, Waltham, MA, USA), mzCloud (ddMS2)(www.mzcloud.org) and ChemSpider [28]. In the instance of one metabolite corresponding to multiple molecular ion peaks, the peak with the highest peak area and highest frequency of retention time was selected for metabolite annotation. Base peak chromatograms were generated using Compound Discover v. 3.3 and data was further processed to report all main peaks with manual curation of annotations. Compounds which remained unannotated after curation were queried using GNPS foodMASST2 [29,30].

### *Ex vivo* microbial culturing experiments

**Fig 1:**
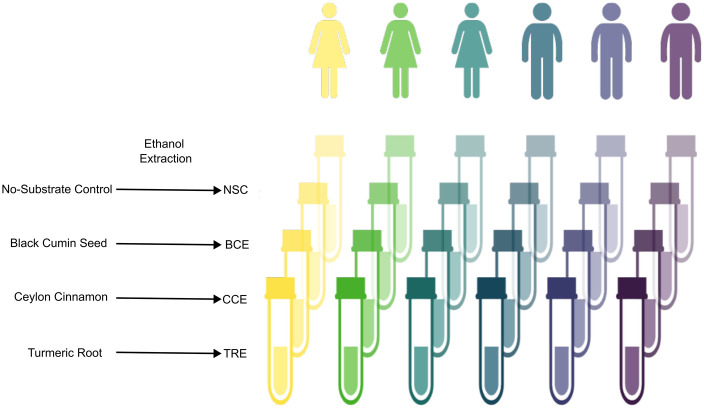
Diagram of experimental design.

Fecal samples were collected from 6 healthy adult donors, 3 males and 3 females between the ages of 25–40 with a BMI between 20–25. Donors were non-smokers, drank less than 3 servings of alcohol/day, had no gastrointestinal disorders or cancers, were not on medications to treat psychological disorders or allergies, and had no anti-/pre-probiotics for at least three months before donations. Donors provided consent prior to collection and IRB approval was given by the Ethics Committee of the University Hospital Ghent (BC-09977). Immediately following collection, feces were homogenized to create a slurry in phosphate buffer. Incubations were conducted for 48 hours using the *ex vivo* SIFR® technology as described previously (Cryptobiotix, Ghent, Belgium) [31,32]. Fecal sample of each donor was used for 4 incubations, non-substrate control (NSC) which is the background media only, in this case medium M0003 (Cryptobiotix, Ghent, Belgium), and then either of the 3 spice extracts at 3g/L to simulate a 3g per day human dose: turmeric root extract (TRE), ceylon cinnamon extract (CCE), or black cumin extract (BCE) (Fig 1). This design used a higher dose than would be typically used as a supplement to allow for better analysis of potential effects using a 48-hour model.

### Shotgun sequencing and cell count analysis

As described previously, total bacteria cell counts were determined using a BD FACS Verse flow cytometer (BD, Erembodegem, Belgium) and analyzed using FlowJo v. 10.8.1 [32].

CosmosID (CosmosID Inc., Rockville, MD, USA) performed the DNA extraction from pelleted bacterial samples using the DNEasy Powersoil Pro Kit (Qiagen, Germantown, MD, USA) and performed shotgun sequencing as described previously using an Illumina HiSeq X platform 2x150bp (Baltimore, MD, USA) with a target depth of approximately 3 million read pairs per sample [33–36].

Preprocessing of raw reads was performed via adapter removal and quality trimming using BBDukv.38.79 with the parameters: (hdist = 1, k = 31, ftm = 5, qtrim = r, trimg = 10) [37]. BBDUk was also used to filter the reads and remove those that mapped to the human genome. Filtered reads were inputted for MetaPhlAn4 v.4.0.6 along with the mpa_vOct22_CHOCOPhlAnSGB_202212 database to execute read-based taxonomic assignment and evaluate relative abundance. HUMAnN v.3.6 was used to generate read-based functional profiles and these were normalized to CPM [38].

### Metabolic analysis

Gas production output was measured by pressure in the headspace of each bioreactor at the beginning and the end (48 hours) of the experiment. A Senseline pH meter F410 (ProSense, Oosterhout, The Netherlands) was used to measure the pH for each culture.

SCFAs were measured as described previously using a diethyl ether extraction method [32,39]. SCFAs that were quantified were: acetate, butyrate, propionate, valerate, and the following branched-chain fatty acids: isocaproate, isobutyrate, and isovalerate. In summary, 0.5 mL of each sample was diluted using distilled water in a 1:3 ratio, 0.5 mL of 48% sulfuric acid (Carl Roth, Karlsruhe, Germany) was added to each sample, and finally sodium chloride (Carl Roth, Karlsruhe, Germany) was added in excess along with 2 mL of HPLC grade diethyl ether (Chem-Lab Analytical, Zedelgem, Belgium) and 0.2 mL of 2-methylhexanoic acid (Sigma Aldrich, Hoeilaart, Belgium) as the internal standard. Following homogenization and separation of the diethyl ether and water layer, the extracts were collected and analyzed using a Trace 1300 chromatograph (Thermo Fisher Scientific, Merelbeke, Belgium) using a Stabiliwax-DA capillary GC column, flame ionization detection and split injector. The temperature profile was set from 110°C-240°C and the injection volume was 1 μL.

### Statistical analysis

R/RStudio (v.4.1.3) was used to conduct statistical analysis using the following packages: vegan (v.2.6−2) [40], ape (v5.6-2) [41], and tidyverse (v.1.3.1) [42]. For statistical analysis of treatment effects, differences between study arms were compared with the NSC using repeated measures ANOVA analysis (based on paired testing). For analysis of microbial composition, three measures were taken, as elaborated by Van den Abbeele et al. (2023) [32]. First, the statistical analysis was performed on log10-transformed values. Second, a value of a given taxonomic group below the limit of detection (LOD) was considered equal to the overall LOD. Finally, a threshold was set to retain the 100 most abundant species in the analysis, to avoid excessive p-value corrections.

## Results

### Untargeted metabolomics analysis of spice extracts

Extracts of black cumin seeds (BCE), turmeric root (TRE) and Ceylon cinnamon (CCE) were subject to untargeted metabolomics analysis to determine key chemical compounds, the 20 most abundant metabolites for all were determined and are shown in Supplemental Tables 1 & 2. Base-peak chromatograms were created and manually annotated to further analyze these extracts (S1 and S2 Figs), and S3-S8 Tables annotate these chromatograms.

BCE selected compounds of interest from the positive mode were choline, indole, cinnamic acid, leucylproline, and oleic acid. From the negative mode analysis of BCE, selected compounds of interest were some sugars, including trehalose, raffinose and lyxose, along with other compounds like gluconic, linoleic acid, and azelaic acids. These results, especially including oleic acid and linoleic acid are similar to those found previously by other preparations of ethanolic extracts of black cumin [7]. For TRE, selected compounds of interest from the positive mode were choline, curcumin, demethoxycurcumin, and turmerone, whereas for the negative node results there were many acids including lactic acid, fumaric acid, isoferulic acid, succinic acid and pyruvic acid. These TRE results mimic those found previously by different ethanolic extracts of turmeric root [43,44]. For CCE, some compounds of interest from the positive node analysis are cinnamaldehyde, piperine, sinomenine, styrene, and choline; whereas for the negative node results there were many acids present, similarly to the other spice extracts, including oleic acid, trans-cinnamic acid, pyruvic acid, fumaric acid, and salicylic acid. Many of these compounds, especially including styrene, cinnamaldehyde, and trans-cinnamic acid have also been found in other preparations of Ceylon cinnamon extracts [45].

### High-level community composition changes with the addition of spice extracts

**Fig 2:**
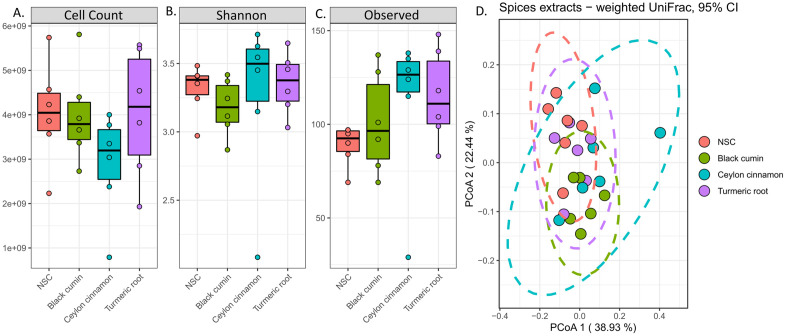
Alpha and beta diversity of *ex vivo* cultures. A) cell counts determined by flow cytometry; **b)** Shannon’s diversity index; c) observed ASVs, a measure of species richness; d) weighted UniFrac visualized using PCoA. PERMANOVA was used to determine statistical significance for all measures.

Cell counts were determined (Fig 2a) at 48 hours of incubation with BCE, TRE, CCE, or the non-substrate control (NSC). This figure demonstrates that CCE decreased the gut microbiota population, whereas BCE and TRE did not when compared with the NSC. However, these results were not statistically significant.

Next, we used the sequencing information to perform alpha diversity analysis using Shannon’s diversity index and observed species (richness) as measures. In terms of Shannon’s diversity, there were no significant changes in diversity regardless of treatment (Fig 2b), though BCE does appear to reduce it slightly. BCE treatment did not deviate from NSC in terms of species richness (Fig 2c), however TRE and CCE appeared to increase species richness of the gut microbiotas compared with NSC. This is surprising given that CCE appeared to decrease cell count compared with the NSC, however, neither of these changes were statistically significant. Finally, weighted UniFrac analysis was used to observe changes in beta diversity in Fig 2d and no significant differences were found between treatments. Ultimately, TRE was the most similar to NSC. BCE-treated samples were the least diverse, indicating that BCE treatment changed the donor gut microbiotas to be more similar to each other. Gut microbiotas treated with CCE had the most diversity between samples, but these changes were not large enough to cause significant separation from NSC. These findings demonstrate that these spices do not significantly change the overall community structure, however it is imperative to explore the gut microbiota at a larger phylogenetic resolution to fully understand treatment effects.

### Taxon changes are most apparent with CCE and BCE

**Fig 3:**
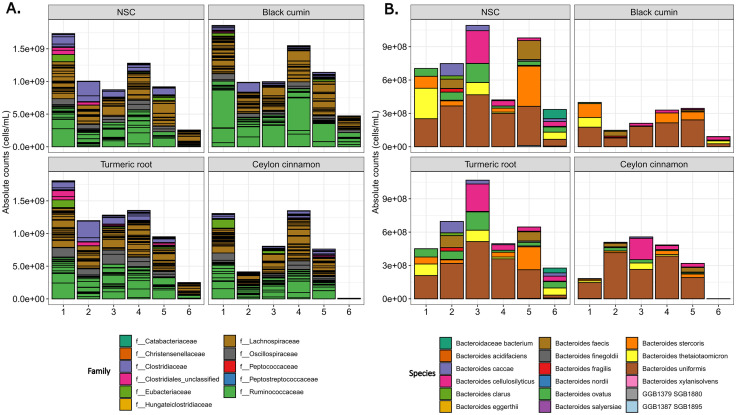
Taxonomic changes based on relative abundance. a) Clostridiales order; b) *Bacteroidaceae* family. MaAsLin2 was performed to determine statistical significance (adjusted p < 0.05).

**Fig 4:**
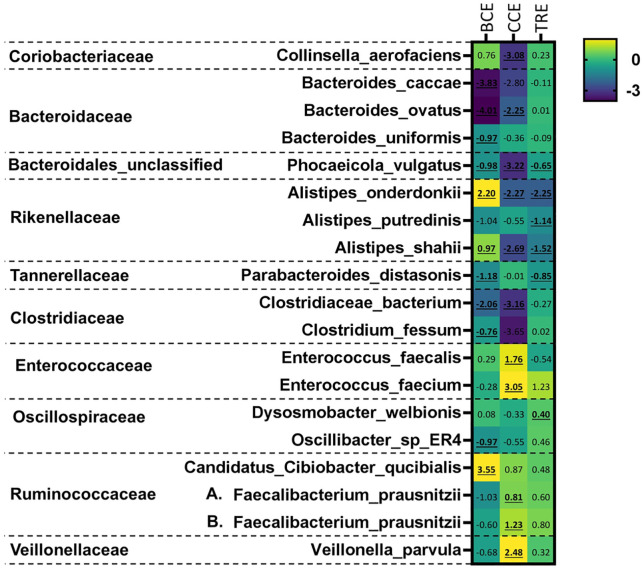
Heatmap of species that changed significantly with treatment. Illustration of the log2 (abundance ratio of treatment/NSC) at the species level, taking into consideration the 100 most abundant species. Repeated measures ANOVA were performed based on log10-transformed absolute levels and significance (non-adjusted p-value < 0.05) is shown in bold and underlined. **A.** *F. prausnitzii* is from the SBG15318 group; **B.** *F. prausnitzii* is from the SGB15342 group.

Relative abundance plots at the phylum level (S3 Fig) demonstrate that the *ex vivo* communities developed over 48 hours are dominated by Actinobacteria, Bacteroidetes, Firmicutes, and Proteobacteria, as is expected in human gut microbial communities. Normalized relative abundance measures were used to determine whether the three spice extracts had different impacts on specific taxa and significance was determined using Tukey’s multiple comparisons of means. At the family level (S4 Fig), there was a significant decrease (q < 0.05) in the *Bacteroidales*_unclassified, *Desulfovibrionaceae*, *Rikenellaceae*, and *Coriobacteriaceae* families with CCE treatment. There was a significant increase (q < 0.0005) in the *Erysipelotrichaceae* family with both CCE and TRE treatment. Some taxa were singled out due to significant changes. For the order Clostridiales, shown in Fig 3a, there was a significant decrease in the family *Clostridiaceae* with CCE treatment (q < 0.05). *Oscillospiraceae* also decreased with CCE treatment, but not significantly once the p-value was adjusted. All other families in the Clostridiales order did not change significantly with any treatment (S9-S10 Tables). B*acteroidaceae* decreased in abundance with CCE and BCE treatment, though again this was not a statistically significant change. However, one taxon within *Bacteroidaceae* did see a significant change in abundance (Fig 3b). *Bacteroides ovatus* significantly decreased in abundance (q < 0.05) with BCE and CCE treatment. Similarly to the order level, at the genus level there was only one taxa that differed significantly by treatment, (“*Clostridiaceae*_unclassified”) at q < 0.05, therefore the species level was of more interest. Due to high interpersonal differences, unadjusted p-values were also used to determine significant changes (p < 0.05) in species abundance between treatments (Fig 4). This revealed that particularly BCE and CCE strongly modulated the abundance of species compared with NSC, with TRE treatment exerting milder effects.

With CCE treatment, there was a significant increase in the health related *Faecalibacterium prausnitzii* along with *Enterococcus* species, specifically *Enterococcus faecalis* and *Enterococcus faecium*, and *Veillonella parvula*. CCE treatment also caused a significant decrease in abundance compared with NSC of the following species: *B. ovatus, Phocaeicola vulgatus, Alistipes onderdonkii,* and *Clostridiaceae bacterium*.

Further, BCE very specifically enhanced *A. onderdonkii*, *Alistipes shahii* and Candidatus *Cibiobacter qucibialis*, while significantly decreasing *B. ovatus*, *Bacteroides caccae*, *Bacteroides uniformis*, *P. vulgatus*, *C. bacterium*, and *Clostridium fessum*.

Finally, upon TRE treatment, there was a significant decrease in *Parabacteroides distasonis, A. shahii* and *A.onderdonkii*. However, there were also some smaller, non-statistically significant changes in health-associated species, including an increase in beneficial species, such as *Dysosmobacter welbionis* and *Faecalibacterium prausnitzii*. Overall, CCE and BCE treatment thus most strongly affected specific gut microbial species, with TRE treatment causing fewer significant changes in the abundance of specific taxa than the other two spice extracts.

### Fermentative outputs change most notably with the addition of BCE

**Fig 5:**
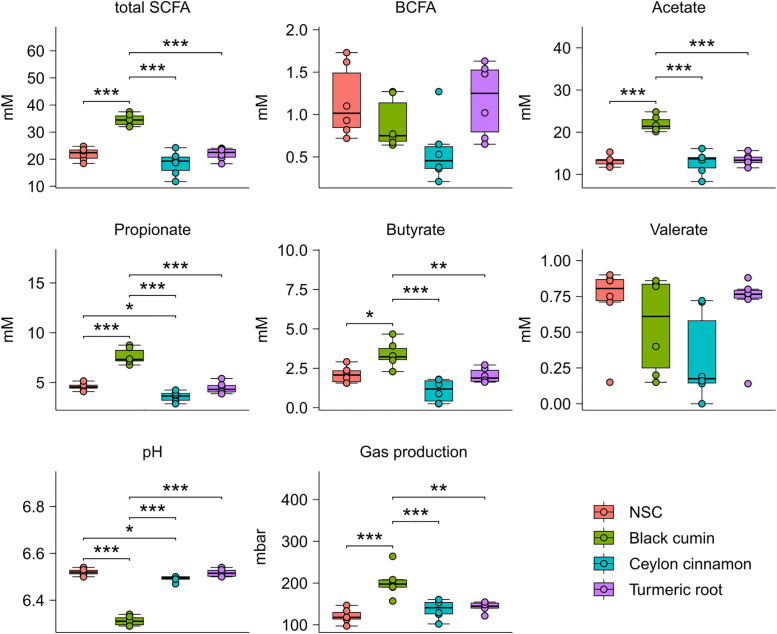
Targeted metabolomics, gas production, and pH. Concentration of SCFAs (mM), BCFAs (Branched-Chain Fatty Acids) (mM), gas production (mbar); and pH. Significance was determined using ANOVA (*** p < 0.0005).

Finally, an analysis of the metabolic outputs produced by the gut microbiomes was performed to understand how each treatment may change the functionality of the microbial communities (Fig 5). Total SCFA concentration remained the same for TRE and CCE treatment when compared with NSC, however, BCE treatment increased total SCFA production. BCE treatment did not produce more branched-chain fatty acids than NSC, neither did TRE, however, CCE reduced BCFA production. Acetate, propionate, and butyrate are three of the most abundant SCFAs produced by the gut microbiota, and in all three cases, only BCE increased their production. Valerate, another common but less abundant SCFA, had no significant changes with any treatment compared with NSC. Gas production and pH are additional ways to quantify the fermentative output of these three treatments. Gas production increased and pH decreased with BCE; whereas TRE and CCE do not deviate from NSC for either measure. This agrees with the observed trend where BCE increases SCFA production, this in turn decreases the pH of the culture and increases gas production.

Analysis of the metabolic outputs normalized to cell count performed by flow cytometry was also performed (S5 Fig) with significance determined using ANOVA. Using this method, we observed that BCE does indeed continue to increase SCFA concentration when compared with NSC, however that increase in only statistically significant by cell count for butyrate. This method also demonstrates that the antimicrobial effects found with CCE treatment (Fig 2a) are likely responsible for the decrease found in BCFA production when compared with the NSC, however this decrease was not statistically significant in either Fig 5 or S5 Fig.

## Discussion

The three spices discussed here, black cumin, turmeric root, and Ceylon cinnamon, all reportedly have beneficial effects on gastrointestinal health, yet little has been done to determine their effect on the human gut microbiota, which is critical for gut health. This study used shotgun sequencing and targeted metabolomics to understand how each of the spice extracts impacts the gut microbiota and whether those impacts are responsible for any of their stated health benefits.

In terms of overall community structure and diversity, BCE, TRE, and CCE did not significantly change the gut microbiota. Although not statistically significant, CCE treatment showed a small reduction in cell count compared to NSC, which is in line with previous studies showing that cinnamaldehyde, one of the key components of the extracts used here, had antimicrobial effects both *in vitro* and *in vivo* [21,46]. CCE treatment modified the gut microbiota by decreasing the abundance of *Clostridiaceae* and *Oscillospiraceae*, both of which are associated with increased inflammation [47,48]. Importantly, CCE increased the abundance of a key player with anti-inflammatory properties, *F. prausnitzii,* which has demonstrated protective effects against colitis models [49,50]. There was also an increase in *Enterococcaceae* species, which are lactate producers and *V. parvula*, which is a lactate-consumer and producer of acetate and propionate, a corresponding effect that has been previously reported [51–53]. The combination of the decrease in inflammatory-associated bacteria, the increase in anti-inflammatory bacteria, and the increase in SCFA producing bacteria are a likely explanation for the reported positive effects of Ceylon cinnamon in treating gastrointestinal distress.

BCE also caused some changes to particular taxa in the gut microbiota, specifically of two members. The first, *Candidatus Cibiobacter qucibialis,* increased significantly with BCE treatment. This bacterium is not well described in the literature but has been found to be depleted in patients with irritable bowel disease [54]. *B. ovatus*, which decreased in abundance with both BCE and CCE treatment, is generally thought to be a beneficial member of the gut microbial community and has been shown in previous studies to increase SCFA abundance [55]. However, this beneficial effect is greatly dependent on the strain, which is not within the parameters of this study. The only significant increase found with BCE treatment was that of *A. onderdonkii*, a member of the *Alistipes* genus that is resistant to bile acids, that has also been shown to reduce proliferation of pancreatic cancer cells *in vitro* [56,57]. The most interesting effect of BCE treatment is its ability to greatly increase SCFA abundance, and subsequently significantly decrease the pH. The increase in SCFA producing bacteria and the subsequent increase in the production of acetate, propionate, and butyrate could be an explanation for the reported beneficial effects of black cumin seeds on gastrointestinal health. Cinnamic acid, one of the key metabolites found in the BCE using untargeted metabolomics, is also known to promote the production of SCFAs, particularly acetate, propionate, and butyrate [58]. These findings indicate that the metabolites remaining in BCE have prebiotic effects on these gut microbial communities, and that these metabolites are not found in such high quantities within CCE or TRE, which could explain why they do not see a similar increase in SCFA production.

TRE treatment caused a significant decrease in two *Alistipes* species that have been previously reported as both beneficial and as harmful to health, depending on the study [56]. However, it caused an increase in *F. prausnitzii* and *D. welbionis*, both of which have demonstrated beneficial effects on gut health. *F. prausnitzii* was described in more detail above, but *D. welbionis* has been shown to have anti-inflammatory activity and to counteract obesity development caused by diet [59]. TRE did contain high levels of curcumin, which is thought to be one of the main contributors to the beneficial health effects of turmeric [60]. However, it is worth noting that those studies that saw the most significant change in the gut microbiota with turmeric root did not use an extract, but powdered turmeric, therefore it is probable that the delivery method of turmeric root makes a substantial contribution to the beneficial effects of turmeric [18].

In conclusion, with this *ex vivo* study, we found that BCE, CCE, and TRE did not have a substantial effect on the gut microbiota *ex vivo*. In fact, TRE had very small effects on the gut microbiota, whereas CCE and BCE had some effects in terms of increased or decreased abundance of particular taxa. BCE, however, caused a significant increase in production of SCFAs, signifying that this extract caused the largest increase in metabolic function of the gut microbiota.

## Supporting information

S1 Fig Positive-mode chromatograms from each extract. A) BCE; B) TRE; C) CCS.
(TIF)

S2 Fig Negative-mode chromatograms from each extract. A) BCE; B) TRE; C) CCS.
(TIF)

S3 Fig Phylum level normed relative abundance. (TIF)

S4 Fig Normalized significant family changes in abundance. A) *Erysipelotrichaceae* significantly increased with CCE treatment; B) *Bacteroidales_unclassified* significantly decreased with CCE treatment; C) *Desulfovibrionaceae* significantly decreased with CCE treatment; D) *Rikenellaceae* significantly decreased with CCE treatment; E) *Coriobacteriaceae* significantly decreased with CCE treatment; F) *Erysipelotrichaceae* significantly increased with TRE treatment.
(TIF)

S5 Fig Targeted metabolomics normalized to cell count. Total SCFA, BCFA, and specific SCFAs, along with gas production normalized to flow cytometry data. Significance determined using ANOVA (* = p < 0.05).
(TIF)

S1 Table Untargeted metabolite analysis positive mode. (XLSX)

S2 Table Untargeted metabolite analysis negative mode. (XLSX)

S3 Table Positive Mode BCE untargeted analysis top 20 metabolites. (XLSX)

S4 Table Positive Mode TRE untargeted analysis top 20 metabolites. (XLSX)

S5 Table Positive Mode CCE untargeted analysis top 20 metabolites. (XLSX)

S6 Table Negative mode BCE untargeted analysis top 20 metabolites. (XLSX)

S7 Table Negative mode TRE untargeted analysis top 20 metabolites. (XLSX)

S8 Table Negative mode CCE untargeted analysis top 20 metabolites. (XLSX)

S9 Table Family-level significance table with adjusted q values. (XLSX)

S10 Table Metabolomic raw data file. Contains data from SCFA, BCFA, gas production, and cell count analyses.
(XLSX)
