# Supplementary figures and images for: The ex vivo effects of ethanolic extractions of black cumin seed, turmeric root, and Ceylon cinnamon bark on the human gut microbiota
Source: PLoS One. 2025 Dec 4;20(12):e0334824. doi: 10.1371/journal.pone.0334824 (PMC12677443; doi:10.1371/journal.pone.0334824)

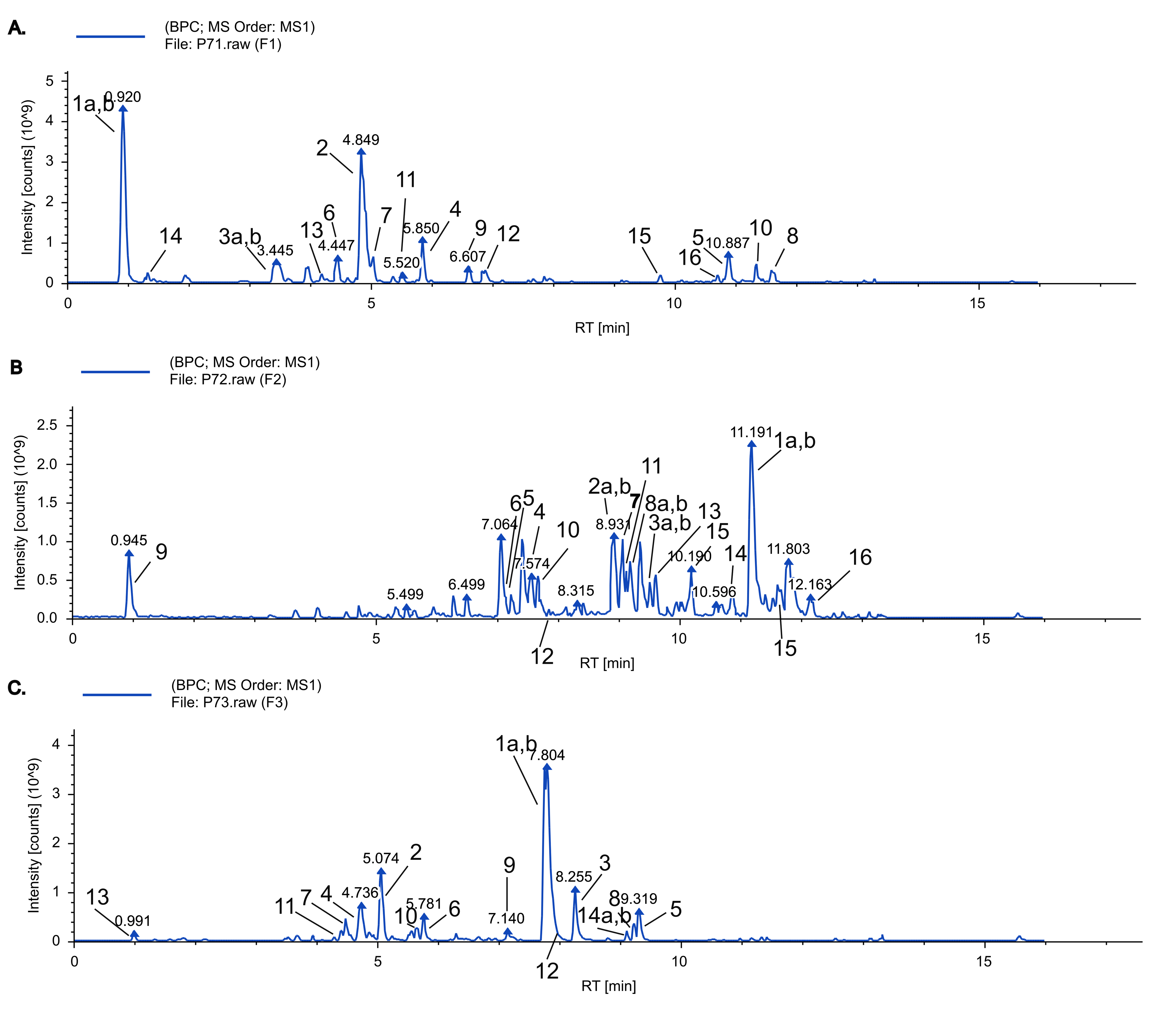

Supplement: S1 Fig — A) BCE; B) TRE; C) CCS. (TIF) [file pone.0334824.s001.tif]

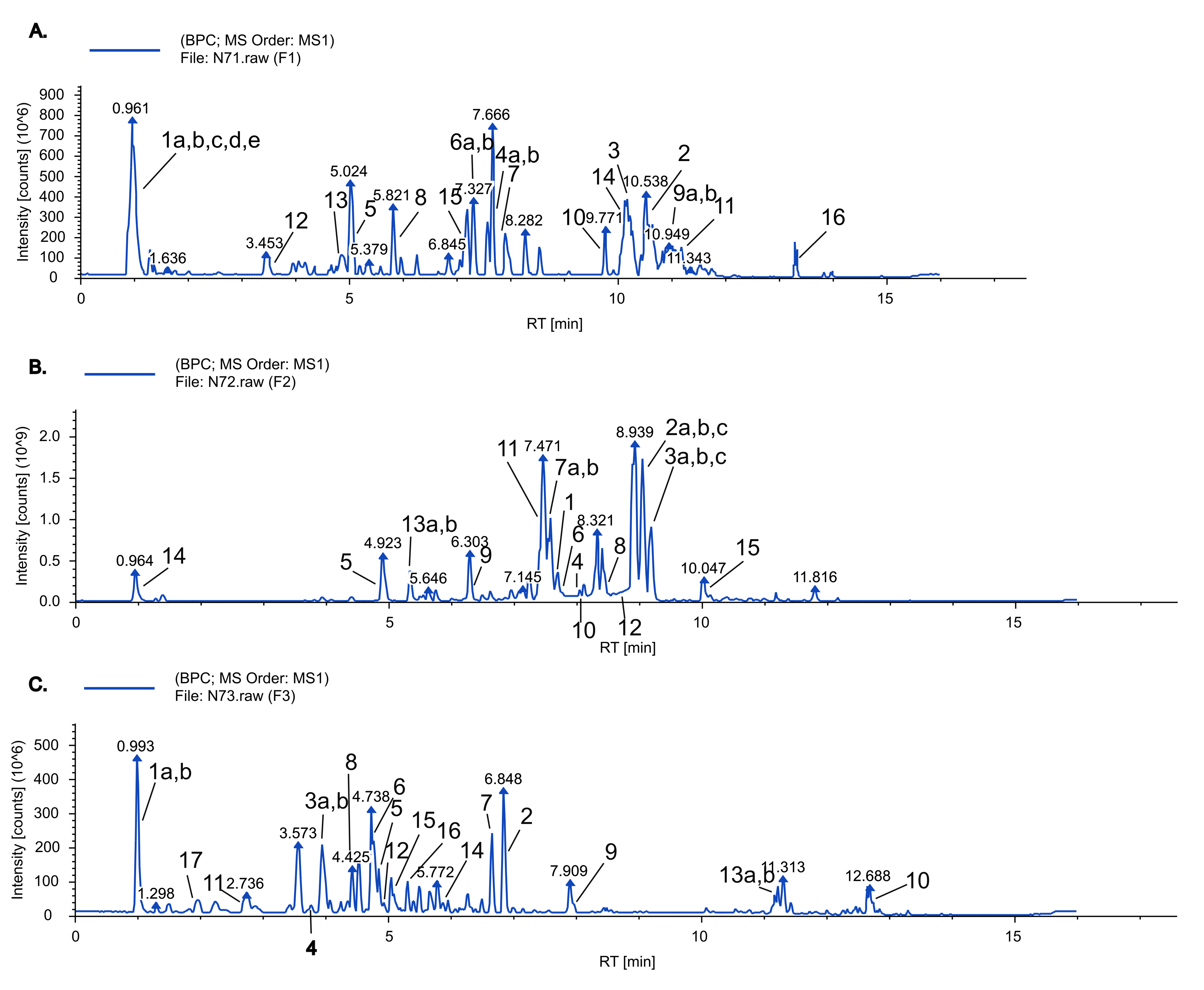

Supplement: S2 Fig — A) BCE; B) TRE; C) CCS. (TIF) [file pone.0334824.s002.tif]

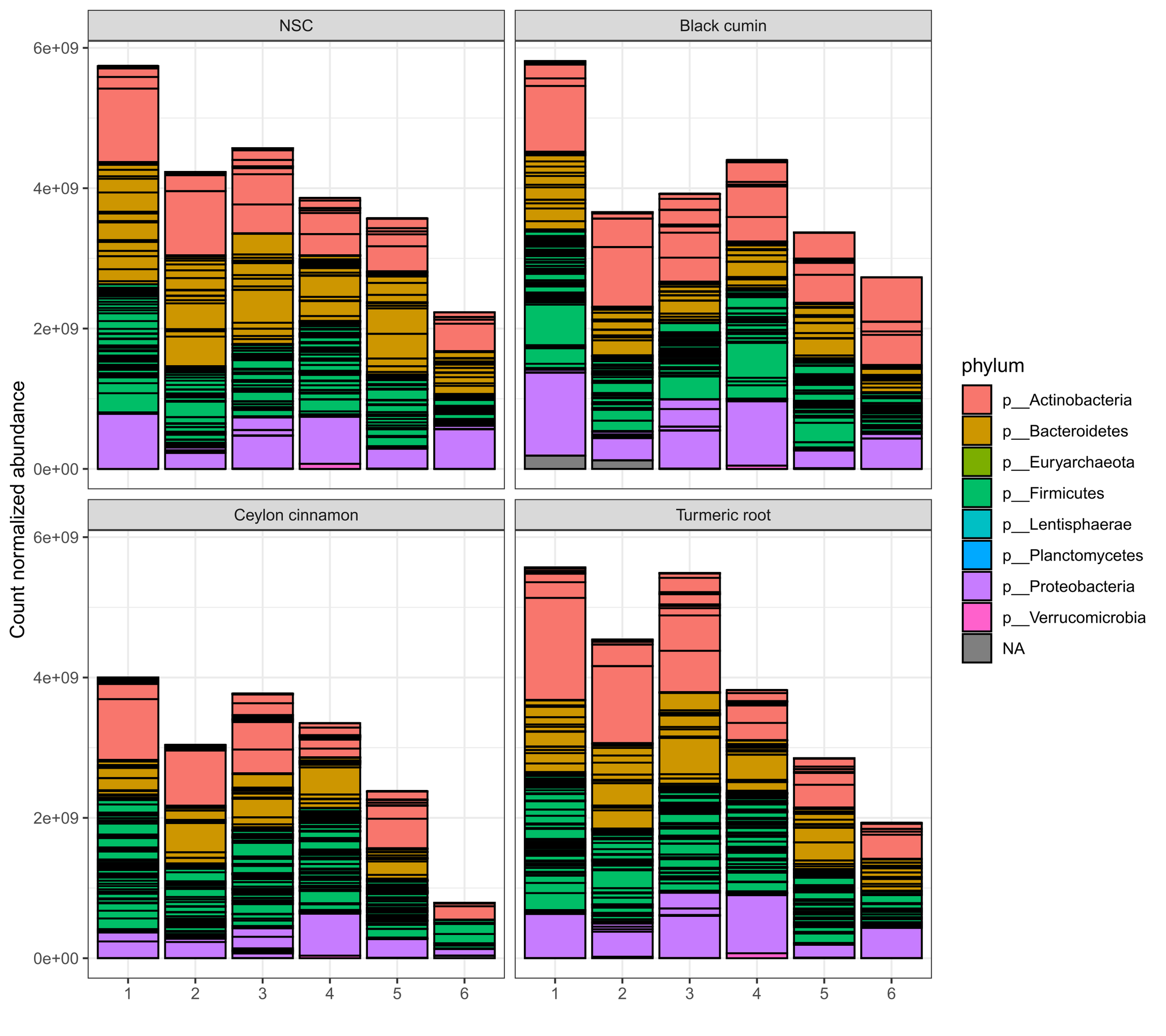

Supplement: S3 Fig — (TIF) [file pone.0334824.s003.tif]

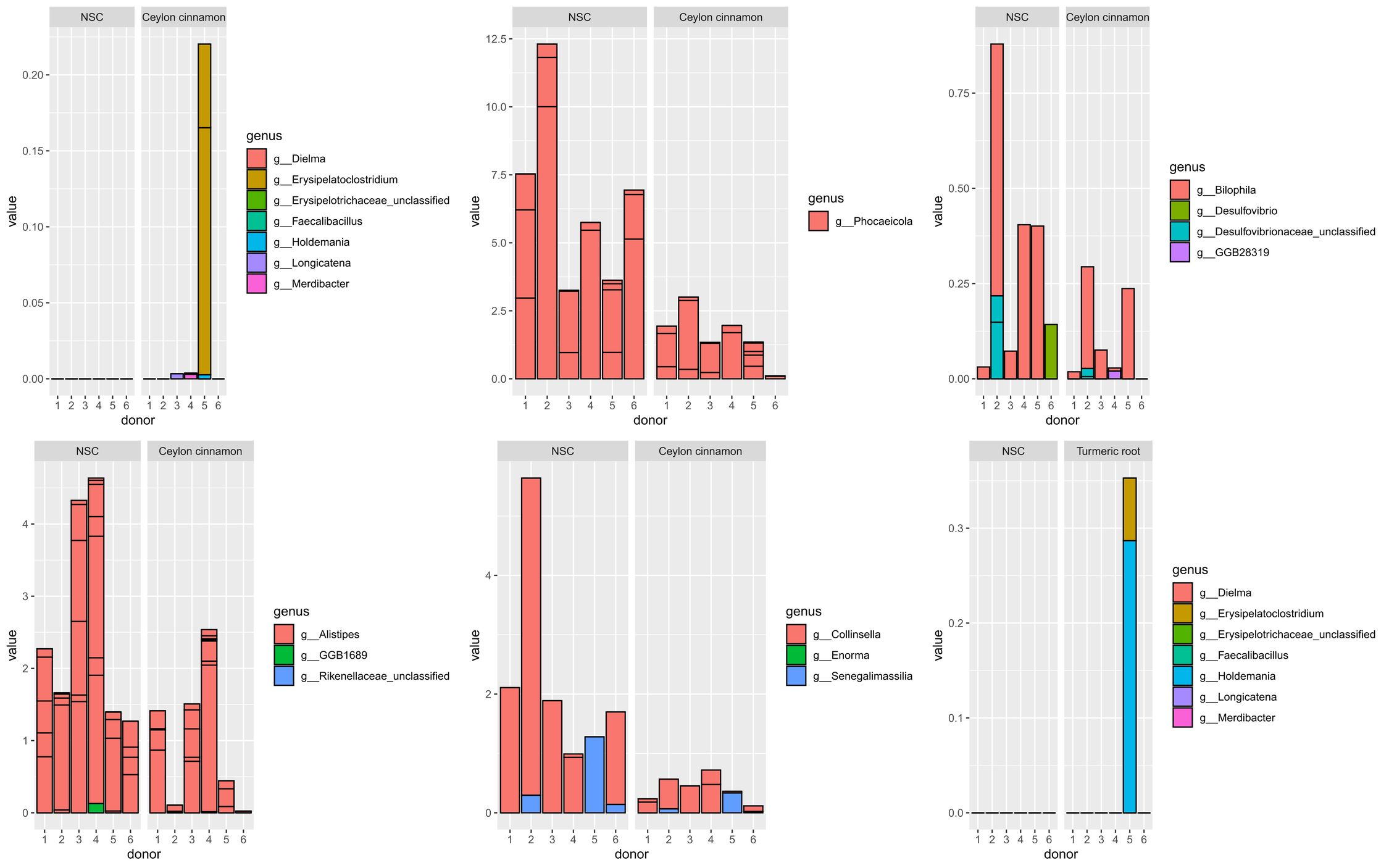

Supplement: S4 Fig — A) Erysipelotrichaceae significantly increased with CCE treatment; B) Bacteroidales_unclassified significantly decreased with CCE treatment; C) Desulfovibrionaceae significantly decreased with CCE treatment; D) Rikenellaceae significantly decreased with CCE treatment; E) Coriobacteriaceae significantly decreased with CCE treatment; F) Erysipelotrichaceae significantly increased with TRE treatment. (TIF) [file pone.0334824.s004.tif]

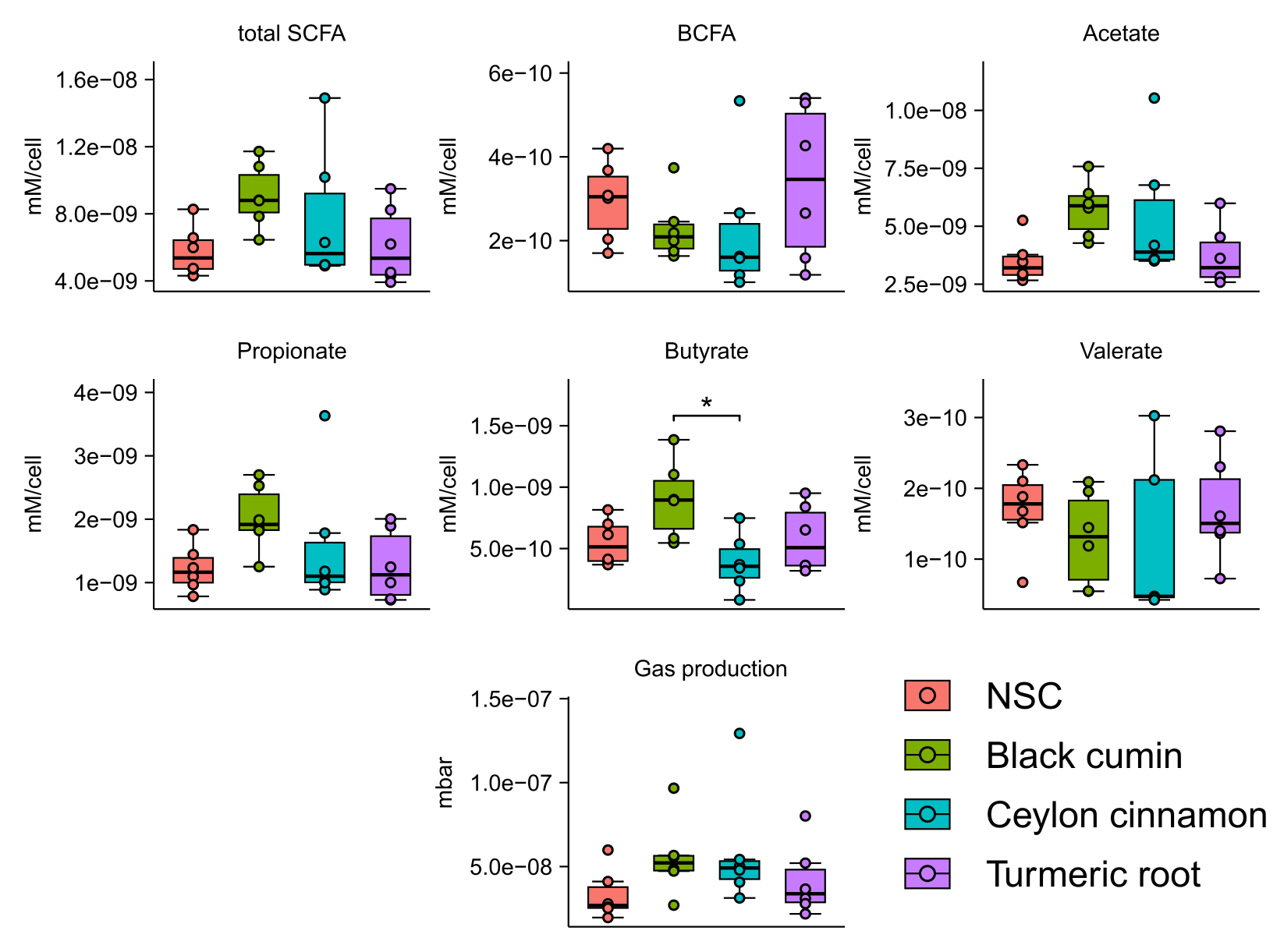

Supplement: S5 Fig — Total SCFA, BCFA, and specific SCFAs, along with gas production normalized to flow cytometry data. Significance determined using ANOVA (* = p < 0.05). (TIF) [file pone.0334824.s005.tif]
